# Burnout dimensions and associated risk factors in medical students: A cross-sectional study in Serbia

**DOI:** 10.1371/journal.pone.0344119

**Published:** 2026-03-06

**Authors:** Irena Ilic, Ivana Zivanovic Macuzic, Milena Ilic, Ana Ravic-Nikolic, Vesna Milicic

**Affiliations:** 1 Faculty of Medicine, University of Belgrade, Belgrade, Serbia; 2 Department of Anatomy, Faculty of Medical Sciences, University of Kragujevac, Kragujevac, Serbia; 3 Department of Epidemiology, Faculty of Medical Sciences, University of Kragujevac, Kragujevac, Serbia; 4 Department of Dermatovenerology, Faculty of Medical Sciences, University of Kragujevac, Serbia; Takushoku University, JAPAN

## Abstract

**Introduction:**

Burnout syndrome presents a serious problem for medical students in their training and professional career. The aim of this study was to investigate the dimensions of burnout syndrome and associated risk factors among medical students in Serbia.

**Methods:**

This was a cross-sectional study. Prevalence of high risk of burnout in medical students was determined following the three-dimensional criteria, based on high levels of emotional exhaustion, cynicism, and low academic efficacy, according to the Maslach Burnout Inventory Student Survey. Odds ratios (OR) with corresponding 95% confidence intervals (95% CI) were determined using multivariate logistic regression analysis.

**Results:**

Risk for high level of all three burnout dimensions (emotional exhaustion, cynicism, and academic inefficacy) was increasing with higher frequency of alcohol consumption (p for trend = 0.008, p = 0.030, p = 0.040, respectively) in medical students. Risk for high level of emotional exhaustion was associated with clinical training level of studies (OR = 1.82, 95% CI = 1.38–2.80, p = .007) and use of sedatives (OR = 3.08, 95% CI = 1.29–7.38, p = 0.011). Length of study >6 years and use of sedatives were associated with high level of cynicism in medical students (OR = 1.98, 95% CI = 1.08–3.62, p = 0.027; OR = 3.19, 95% CI = 1.31–7.75, p = 0.010, respectively). Higher grade point average (>8) was most common in medical students with lower risk of academic inefficacy (OR = 0.65, 95%CI = 0.44–0.95, p = 0.026). Positive personal medical history (presence of any chronic disease) was frequently reported in medical students who had a high risk of academic inefficacy (OR = 2.45, 95% CI = 1.30–4.61, p = 0.006).

**Conclusion:**

Some characteristics of medical students were associated with high risk of burnout, indicating the need for psychological support aimed at providing appropriate help and assistance to medical students.

## Introduction

Burnout syndrome is defined as a “psychological syndrome that implies emotional exhaustion, cynicism and a feeling of little personal accomplishment, in people who perform their work in dynamic relationships with other people, in professionally demanding situations” [1]. Symptoms of emotional exhaustion are central and accompanied by depersonalization and a feeling of reduced personal accomplishment [2,3]. Emotional exhaustion is marked by a feeling of emotional overspending due to work. Depersonalization is characterized by emotional indifference and dehumanization of those receiving services. Reduced personal achievement is reflected in feelings of professional stagnation, incompetence and lack of fulfillment. Emotional exhaustion refers to feeling emotionally “stretched” and exhausted from contact with other people and it represents the “stressful” component of burnout. Depersonalization (i.e., cynicism, dehumanization), representing the interpersonal component of burnout, involves emotional distance in professional roles and refers to an emotionless and indifferent attitude towards those receiving help, which can turn into rude or even inappropriate behavior toward patients, as well as other people at work [2,4]. Reduced personal accomplishment, the self-evaluative component of burnout, refers to the perceived decline in skillfulness and successful achievement at jobs that involve working with people, which can lead to a profound sense of failure, due to persistent relational stressful events at workplace [2–4].

Due to intense, strenuous studying and psychophysical exhaustion, medical students may experience a certain degree of burnout syndrome, as academic stress. Burnout can have serious professional and personal consequences – it can affect students’ attitudes toward a career in medicine, and lead to thoughts of dropping out, depression, alcohol and drug abuse and suicide [5,6]. Among medical students, burnout often arises from the intense stress they have experienced and the expectations of high standards placed on individuals involved in careers in helping professions [2,7]. The medical school curriculum and some components of training/practice can lead to chronic stress and burnout in some medical students [8,9]. Some studies report that, in the developed world, at least every second medical student experiences some form of burnout during their studies [10–13].

A meta-analysis that pooled data from 24 studies, comprising 17,431 medical students, found high prevalence of emotional exhaustion, depersonalization and personal accomplishment (40.8%, 35.1% and 27.4%, respectively) [11]. More recently, one larger meta-analysis showed that the frequency of emotional exhaustion, depersonalization, and personal accomplishment in medical students was 38.08%, 35.07%, and 37.23%, respectively [14].

Some studies have reported associations between burnout prevalence and gender [14,15], year of study [16,17], personal life events and the learning environment [18], but results were not consistent [11,19,20]. Additionally, some authors found no association between burnout and marital status, smoking, presence of chronic diseases in personal health history or completed high school [15]. Among students, findings regarding the association between burnout dimensions and gender are conflicting. While some authors [21] reported a significant association between male gender of students and depersonalization, other authors found no significant association [14,18].

Studies on burnout syndrome among medical students in Serbia are relatively scarce, and only a few of have utilized the validated MBI-SS questionnaire [19]. Existing research shows that burnout is prevalent in medical students in Serbia, with studies reporting high rates of emotional exhaustion, cynicism, and academic inefficacy [20,21]. Factors contributing to this risk include older age, alcohol use, stress related to training conditions, specific factors such as male gender and higher study years, but these findings have not been consistent across studies [20–22]. This study aimed to assess the prevalence of high levels of emotional exhaustion, cynicism, and academic ineffectiveness among medical students in Serbia, as well as their association with characteristics of medical students.

## Methods

### Setting

This study was conducted at the Faculty of Medical Sciences, University of Kragujevac, Serbia – the youngest state medical faculty in the country, founded in 1977. The Integrated Academic Studies of Medicine study program (formally and structurally harmonized with the European accreditation standards in 2005) is designed to provide students with complete and comprehensive education and up-to-date scientific and professional knowledge and skills in the field of medicine. The study program lasts six years and leads to the academic professional title of doctor of medicine. Medical studies are free, with approximately 80% of medical students receiving a stipend during their studies. The amount of this state stipend that almost all students receive for the most part covers the compensation for meals in student restaurants and accommodation in student dormitories. During the program, medical students have practical classes in small groups up to a maximum of 6 students for clinical training and up to a maximum of 12 for preclinical courses.

At the Faculty of Medical Sciences in Kragujevac, each medical student is assigned a mentor from the teaching staff. Mentors have weekly (and more frequent if needed) meetings of the entire assigned mentor group to consider and discuss all matters related to attending classes, exams and any possible difficulties during the studies. Each mentoring group typically consists of 6 students, who maintain contact with the mentor via email, phone, or in-person conversation at meetings. Medical students also participate in the student parliament and the sports club, where they can engage in various sports activities. Systemic improvements are continuously being implemented, including better financial support and enhanced institutional resources to facilitate student participation in congresses, sports festivals, international exchanges, etc. The mentor is assigned to each medical student from the outset of their studies and is obliged to monitor their progress in studies throughout the course. This mentoring approach is intended to provide a supportive environment that helps students address the significant demands of medical education, while fostering a sense of togetherness and solidarity within the student community.

### Study design

This study employed a cross-sectional design. Self-reported data were collected through questionnaires that all medical students were given during classes, accompanied by a cover letter containing study information and consent form.

### Study population, study sample and simple size calculation

All students enrolled as regular students at the study year at medical faculty were invited to participate in the study. Students from all six years of medical studies who voluntarily provided signed informed consent to participate were included in the study. Inclusion criteria were: age > 18 years, regular student status, attendance at lectures, signed written informed consent to participate and lack of exclusion criteria. Exclusion criteria were: age < 18 years and non-attendance at regular lectures.

The sample size was calculated using the Epi Info StatCalc software (Centers for Disease Control and Prevention, Atlanta, Georgia, USA). Based on the population size of 836 students, a 5% margin of error, a 99.99% confidence level and an expected prevalence of 22.6%, the minimum required sample size was determined to be 467 [23]. After adjusting for a potential non-response rate of 15%, the final estimated sample size was 538. Of the 836 eligible medical students, 760 participated in research and completely filled out the questionnaire, yielding a response rate of 90.9%. Among the medical students who did not respond to the survey, the main reason for exclusion from the study was absence from regular classes for 45 students, 12 students refused to participate (most commonly due to a lack of interest in the study), while some respondents did not return the questionnaire or did not complete it during the study recruitment or the questionnaires were not fully completed (a total of 19 students).

### Data collection

In addition to the epidemiological questionnaire (which collected data on socio-demographic characteristics, academic performance, habits, personal medical history, etc.), this study assessed the level of risk for burnout syndrome in medical students using the Maslach Burnout Inventory – Student Survey [24]. The survey was self-reported, anonymous and administered in the paper-and-pencil format. Students filled out the questionnaires in amphitheater and lecture auditoriums at the Faculty of Medical Sciences in Kragujevac from February 15 to March 14 2014. Authors of this manuscript, medical doctors, were present while medical students completed the questionnaires. Study participants had about 15(± 5) minutes to fill out the survey.

### Instruments

The epidemiologic questionnaire included items on student characteristics, such as sex (male/ female), age (years: ≤ 21/ 22–24/ ≥ 25), type of completed secondary school (grammar school/ medical and other schools), marital status (with partner/ without partner), whether they have children (No/ Yes), study financing (state-sponsored/ self-funded), housing (in own home/ with parents/ as subtenants/ in student dormitory), enrolled academic year (I – VI study year), achieved academic results (cumulative total point average grade ≤ 8/ > 8, where grade is the average of the subjects taken according to the European credits scores on a 10-point scale, with a passing score established at 6 points), repeated a year of study (No/ Yes), level of study (medical students of first-third year: preclinical training; students of fourth-sixth year: clinical training), number of exams passed (dichotomized: ≤ 23 exams/ > 23 exams), length of study (dichotomized: ≤ 6 years/ > 6 years), habits (smoking cigarettes, drinking alcohol), personal medical history (presence of any chronic disease), use of sedatives and use of psychoactive substances. Students were considered smokers if they regularly smoked at least a cigarette a day during one year, categorized as current smokers if they smoked at least one cigarette every day during the last 12 months, and as former smokers if it has been at least a year since they stopped smoking.

Maslach Burnout Inventory – Student Survey (MBI-SS) is designed to measure burnout level in students and it was developed by Schaufeli et al [24,25]. MBI-SS consists of 15 items that assess three dimensions of burnout: Emotional Exhaustion – EE (5 items), Cynicism – CY (4 items), and Academic Efficacy – AE (6 items). All items refer to the student’s feelings about university, i.e., feelings about their academic work. Students responded to questions by indicating the level to which they agree with each item, scored on a 7-point Likert-type scale ranging from 0 to 6 (0 – never, 1 – several times a year, 2 – once a month, 3 – several times a month, 4 – once a week, 5 – several times a week, 6 – every day), in order to describe how often they felt in a certain way. License to use the MBI-SS was obtained from the current owner (i.e., Mind Garden, Menlo Park, CA, USA). Following this, a linguistic adaptation and validation were done. The validity and reliability of the original three-factor structure of the MBI-SS questionnaire were confirmed in this study: the Cronbach’s α coefficients for EE, CY, and AE were 0.869, 0.856, and 0.852, respectively [19]. In this study, standard Maslach criteria were used (classifying the sample into thirds, with each third indicating “low”, “medium” and “high” burnout) [1–4]. A high level of burnout was defined as: emotional exhaustion over 14 (MBI EE), cynicism over 6 (MBI CY) and low academic efficacy – less than 23 (reverse Аcademic Еfficacy = MBI rAE) [24,25].

### Statistical analysis

Descriptive and analytical statistics were used to analyze the data. Categorical variables were presented as proportions (%), while continuous variables were expressed as means ± standard deviations (SD). Differences between medical students according to burnout dimensions (emotional exhaustion, cynicism, academic inefficacy) were assessed using the chi-square test and t-test. Multivariate logistic regression was used to examine the association between medical students’ characteristics and high risk of burnout syndrome (high risk of emotional exhaustion, cynicism, and academic inefficacy), by determining Odds Ratio (OR) with corresponding 95% Confidence Interval (95% CI).

Only variables (potential correlates) that were significant at the p < 0.05 level based on the chi-square test were entered in the multivariate logistic regression models. Age and year of study were not included in the models due to their collinearity (the Variance Inflation Factor = 4.322), as well as with some variables (level of training, length of study, re-enrollment of study year, number of passed exams, grade point average). Model fit was checked with the Hosmer-Lemeshow test of goodness of fit and Cox and Snell’s and Nagelkerke’s Pseudo R square measures. A test for linear trend in risk was based on the logistic regression model.

All statistical analyses were performed separately for three domains of the MBI-SS. Statistical significance was indicated by p < 0.05. All statistical analyses were done with the SPSS Software (SPSS Inc, version 20, Chicago, IL).

### Ethical considerations

This study is a part of research approved by the Ethics Committee of the Faculty of Medical Sciences, University of Kragujevac (Ref. No.: 01–1176). All participants voluntary provided informed written consent prior to taking part in the study.

## Results

Table 1 presents the triad of symptoms of burnout syndrome. Mean subscale scores were: MBI EE (12.8 ± 7.2; range 0–30), MBI CY (3.6 ± 4.9; range 0–24) and MBI rAE (9.1 ± 8.3; range 0–36).

**Table 1:** A cross-sectional study of burnout syndrome in medical students: mean of MBI-SS subscale scores.

MBI-SS subscales	Mean	SD	Min	Max
**- MBI EE**	12.8	7.2	0	30
**- MBI CY**	3.6	4.9	0	24
- **MBI rAF**	9.1	8.3	0	36

MBI-SS (Maslach Burnout Inventory – Student Survey); MBI EE (Emotional Exhaustion); MBI CY (Cynicism), MBI rAF (reverse Аcademic Еfficacy); SD (Standard Deviation).

According to the risk categories for burnout syndrome, a high risk of emotional exhaustion was recorded in 308 medical students (40.5% of respondents) (Table 2). The cynicism subscale showed a high risk of burnout in 266 participants (i.e., 35.0%). The academic inefficacy subscale showed that 37.7% of students were in the high risk of burnout category.

**Table 2:** Level of risk for burnout syndrome in medical students, by subscales.

MBI-SS subscales	Level of risk	Number (N = 760)	(%)
**MBI EE**	Low risk	256	33.7
	Moderate risk	196	25.8
	High risk	308	40.5
	Total	760	100.0
**MBI CY**	Low risk	258	33.9
	Moderate risk	236	31.1
	High risk	266	35.0
	Total	760	100.0
**MBI rAE**	Low risk	275	36.2
	Moderate risk	198	26.1
	High risk	287	37.7
	Total	760	100.0

MBI-SS (Maslach Burnout Inventory – Student Survey); MBI EE (Emotional

Exhaustion); MBI CY (Cynicism); MBI rAE (reverese Аcademic Еfficacy).

A significant association was found between gender and lower cynicism: compared to men, fewer women showed high levels of cynicism (31.6% vs. 41.3%; p = 0.007) (Table 3). Age significantly correlated with all three burnout dimensions – cynicism and academic inefficacy increased significantly with age (p < 0.001), while emotional exhaustion decreased significantly with age (p < 0.001). The self-funded way of study financing was significantly associated with greater cynicism and greater academic inefficacy (p < 0.05).

**Table 3:** Distribution of medical students with a high burnout score by domain and by sociodemographic characteristics.

		Burnout syndrome – high risk					
		MBI EE		MBI CY		MBI rAE	
Variables		%	*p* ^*, **^	%	*p* ^*, **^	%	*p* ^*, **^
**Gender**							
	Male	37.9		41.3		42.4	
	Female	42.0	0.781	31.6	0.007	35.2	0.052
**Age** (years)							
	≤ 21	39.5		18.6		18.6	
	22-24	45.6		38.2		38.2	
	≥ 25	34.9	0.030	42.2	<0.001	50.4	<0.001
**Average age** (Mean±SD)		23.4 ± 2.5	0.758	24.2 ± 2.7	<0.001	24.5 ± 2.7	<0.001
**Residence**							
	Urban	40.3		35.0		37.6	
	Rural	42.9	0.694	34.9	0.989	39.7	0.743
**Completed secondary school**							
	Grammar school	40.1		36.6		35.5	
	Medical school	40.8	0.855	34.1	0.491	39.0	0.350
**Marital status**							
	With partner	42.2		35.4		38.2	
	Without partner	39.3	0.410	34.7	0.841	37.4	0.832
**Children**							
	No	40.7		34.4		37.4	
	Yes	36.4	0.687	54.5	0.051	50.0	0.230
**Housing**							
	In own home	34.3		35.7		40.0	
	With parents	42.7		39.3		34.8	
	As subtenants	40.8		34.7		39.3	
	In student dormitory	37.7	0.589	20.8	0.028	39.0	0.677
**Study financing**							
	State-sponsored	41.9		32.8		33.0	
	Self-funded	35.5	0.139	42.8	0.018	54.8	<0.001

MBI EE (Emotional Exhaustion); MBI CY (Cynicism); MBI rAE (reverse Аcademic Еfficacy);

SD (Standard Deviation); P (probability: ^*^ χ^2^-test, ^**^ t-test).

Senior academic year, average length of study, training level, and number of exams passed were significantly associated with high emotional exhaustion, high cynicism, and high academic ineffectiveness (p < 0.05) (Table 4). Re-enrollment of a study year was associated with high cynicism and academic inefficacy. A higher GPA was significantly associated with lower cynicism and academic inefficacy (p < 0.05).

**Table 4:** Distribution of medical students with a high burnout score by domain and by academic performance.

		Burnout syndrome – high risk					
		MBI EE		MBI CY		MBI rAE	
Variables		%	*p* ^*, **^	%	*p* ^*, **^	%	*p* ^*, **^
**Study year**							
	1^st^ year	46.2		20.4		17.2	
	2^nd^ year	31.5		13.0		20.7	
	3^rd^ year	57.6		44.4		46.5	
	4^th^ year	47.1		36.2		32.6	
	5^th^ year	34.7		39.3		43.4	
	6^th^ year	32.7	<0.001	44.2	<0.001	51.1	<0.001
**Re-enrollment of the study year**							
	No	42.3		31.1		31.3	
	Yes	35.1	0.086	47.0	<0.001	57.8	<0.001
**Length of study** (years)							
	≤ 6	42.0		31.6		33.3	
	> 6	32.5	0.054	53.8	<0.001	62.4	<0.001
**Length of study, years** (Mean±SD)		4.3 ± 2.5	0.015	5.2 ± 2.7	<0.001	5.5 ± 2.8	<0.001
**Training**							
	Preclinical	45.4		26.4		28.5	
	Clinical	37.6	0.034	40.1	<0.001	43.3	<0.001
**Number of passed exams**							
	≤ 23	45.9		30.3		31.0	
	> 23	33.3	<0.001	41.4	0.002	46.9	<0.001
**Grade point average**							
	≤ 8	37.7		41.7		50.0	
	> 8	42.1	0.228	31.2	0.004	30.8	<0.001
**Grade point average** (Mean±SD)		8.4 ± 0.7	0.949	8.3 ± 0.8	0.014	8.2 ± 0.8	<0.001
**Study per day, hourses** (Mean±SD)		5.0 ± 2.3	0.082	4.7 ± 2.3	0.180	4.8 ± 2.2	0.357
**Sleep per day, hourses** (Mean±SD)		7.0 ± 1.3	0.054	7.1 ± 1.2	0.303	7.1 ± 1.3	0.827

MBI EE (Emotional Exhaustion); MBI CY (Cynicism); MBI rAE (reverse Аcademic Еfficacy);

SD (Standard Deviation); P (probability: ^*^ χ^2^-test, ^**^ t-test).

Compared to non-smokers, cigarette smokers more frequently exhibited high cynicism (40.7% vs. 32.3%; p = 0.023), and high academic inefficacy (43.6% vs. 35.0%; p = 0.022) (Table 5). Medical students who consumed alcohol more frequently showed high emotional exhaustion (p = 0.001), high cynicism (p = 0.003), and high academic inefficacy (p = 0.013). A positive personal medical history (for the presence of any chronic disease) was significantly associated with high academic inefficacy (p = 0.017). Sedative use was significantly associated with high emotional exhaustion (p = 0.026), high cynicism (p = 0.001), and lower academic efficacy (p = 0.022). Substance use showed significant associations with all three dimensions of burnout, with medical students who used psychoactive substances showing high emotional exhaustion (p = 0.046), high cynicism (p = 0.002), and high academic inefficacy (p = 0.029).

**Table 5:** Distribution of medical students with a high burnout score by habits and personal medical history.

		Burnout syndrome – high risk					
		MBI EE		MBI CY		MBI rAE	
Variables		%	*p* ^*, **^	%	*p* ^*, **^	%	*p* ^*, **^
**Cigarette smoking**							
	Never	38.5		32.3		35.0	
	Ever	44.9	0.096	40.7	0.023	43.6	0.022
**Smoking status**							
	Never smoker	38.5		32.3		35.0	
	Former smoker	41.0		39.0		42.0	
	Current smoker	47.6	0.148	42.0	0.067	44.8	0.067
**Age at smoking initiation** (Mean±SD)		18.3 ± 2.5	0.721	18.2 ± 2.7	0.603	18.4 ± 2.7	0.701
**Number of cigarettes/day** (Mean±SD)		13.3 ± 8.4	0.447	13.8 ± 8.6	0.164	14.1 ± 8.9	0.069
**Alcohol consumption**							
	No	42.3		33.1		36.4	
	Yes	39.3	0.416	36.3	0.372	38.7	0.524
**Frequency of alcohol consumption**							
	Non drinkers	42.3		33.1		36.4	
	1-2 times a year	16.7		24.2		28.8	
	1-2 times a month	41.4		34.4		37.4	
	1-2 times a week	50.6		49.4		46.8	
	Every day	40.0	0.001	70.0	0.003	80.0	0.013
**Positive personal medical history**							
	No	39.8		34.9		36.7	
	Yes	52.2	0.097	37.0	0.774	54.3	0.017
**Use of sedatives**							
	No	39.8		33.9		37.1	
	Yes	61.5	0.026	65.4	0.001	57.7	0.022
**Use of psychoactive substances**							
	No	40.2		34.4		37.4	
	Yes	75.0	0.046	87.5	0.002	75.0	0.029

MBI EE (Emotional Exhaustion); MBI CY (Cynicism); MBI rAE (reverse Аcademic Еfficacy);

SD (Standard Deviation); P (probability: ^*^ χ^2^-test, ^**^ t-test).

The risk for high level for all three burnout dimensions (emotional exhaustion, cynicism, and academic inefficacy) in medical students was significantly increased with higher frequency of alcohol consumption (p for trend = 0.008, p = 0.030, p = 0.040, respectively) (Table 6). Risk for high level of emotional exhaustion was associated with clinical training level of studies (OR = 1.82, 95%CI = 1.38–2.80, p = 0.007) and use of sedatives (OR = 3.08, 95%CI = 1.29–7.38, p = 0.011). Among medical students, length of study >6 years was associated with high risk of cynicism (OR = 1.98, 95%CI = 1.08–3.62, p = 0.027). Also, the use of sedatives was linked to high risk of cynicism in study participants (OR = 3.19, 95%CI = 1.31–7.75, p = 0.010). A higher grade point average (>8) was significantly more frequently reported by medical students with lower risk of academic inefficacy (OR = 0.65, 95%CI = 0.44–0.95, p = 0.026). Positive personal medical history (presence of any chronic disease) was significantly more often noted in study participants who had high risk of academic inefficacy (OR = 2.45, 95%CI = 1.30–4.61, p = 0.006).

**Table 6:** High burnout score by domains, according to multivariate logistic regression analysis.

		Burnout syndrome – high risk								
		MBI EE			MBI CY			MBI rAE		
Variables		OR	(95%CI)	*p*	OR	(95%CI)	*p*	OR	(95%CI)	*p*
**Training**										
	Preclinical	1.00^*^								
	Clinical	1.82	(1.38-2.80)	0.007						
**Length of study** (>6 years)					1.98	(1.08-3.62)	0.027			
**Grade point average** (>8)								0.65	(0.44-0.95)	0.026
**Frequency of alcohol consumption**										
	Non drinkers	1.00^*^			1.00^*^			1.00^*^		
	1-2 times a year	0.68	(0.37-1.27)	0.230	0.69	(0.37-1.31)	0.258	0.71	(0.38-1.31)	0.267
	1-2 times a month	1.08	(0.76-1.52)	0.677	1.03	(0.72-1.48)	0.866	1.06	(0.74-1.52)	0.730
	1-2 times a week	1.99	(1.18-3.36)	0.010	1.80	(1.04-3.12)	0.035	1.49	(0.86-2.60)	0.159
	Every day	4.88	(1.17-20.36)	0.030	4.55	(1.06-19.56)	0.042	8.49	(1.62-44.46)	0.011
	*p* for trend			0.008			0.030			0.040
**Use of sedatives** (Yes)		3.08	(1.29-7.38)	0.011	3.19	(1.31-7.75)	0.010			
**Positive personal medical history** (Yes)								2.45	(1.30-4.61)	0.006

MBI EE (Emotional Exhaustion); MBI CY (Cynicism); MBI rAE (reverse Аcademic Еfficacy); OR (Odds Ratio);

95%CI (95% Confidence Interval); 1.00^*^ (Reference category).

## Discussion

This study revealed that high level of all three burnout dimensions (emotional exhaustion, cynicism, and academic inefficacy) was significantly associated with higher frequency of alcohol consumption among medical students. The risk for high level of emotional exhaustion was associated with clinical training level of studies and use of sedatives. High level of cynicism in medical students was significantly associated with the length of study of >6 years and use of sedatives. Medical students who had high risk of academic inefficacy more often reported a positive personal medical history (presence of any chronic disease).

A recent meta-analysis on burnout among medical students reported current prevalence of 40.8% for emotional exhaustion, 35.1% for cynicism, and 27.4% for personal achievement [11]. Similarly, a study in medical and dentistry university students found overall prevalence of burnout syndrome dimensions to be 55.4%, 31.6% and 30.9% for emotional exhaustion, cynicism, and academic efficacy, respectively [26]. In line with these findings, the present study, using the MBI-SS questionnaire, reported prevalence of high risk of 40.5% for emotional exhaustion, 35.0% for cynicism and 37.7% for academic inefficiency. When compared to medical students in Kragujevac, those studying at two universities in Brazil [27,28], the United Kingdom [29], Saudi Arabia [18] and Malaysia [30] exhibited higher prevalence of emotional exhaustion, cynicism and academic inefficacy. In contrast, dentistry students in Turkey [31,32] and medical students in Colombia [33] showed lower prevalence rates. Among general medicine students from four hospitals and universities of medicine in Hungary, 38.6% showed medium or high emotional exhaustion, 34.0% cynicism and 24.0% reduced academic efficiency [34]. Several factors may account for the observed variations in the frequency of dimensions of burnout syndrome in medical students. These include differences across countries, in study populations, culture and socio-economic status. Another explanation could be the high level of heterogeneity in research on medical students’ burnout. For instance, while some studies included medical students from only one or only a few study years, the present study encompassed students from all six years of study. Moreover, studies have employed different instruments for measuring burnout and applied varying cutoff-criteria for burnout. A challenge in comparing findings across studies may also be in the use of different definitions of burnout. While some authors assessed burnout using all three dimensions, others evaluated only two dimensions, or even just one (most commonly emotional exhaustion [35–37]. Additionally, there is a conceptual divergence in how burnout is understood: health professionals often regard burnout as a dichotomous phenomenon, whereas other researchers mostly consider burnout as a continuous phenomenon with ranges from absence to medium to high [35–37]. Further on, variability in sample sizes across studies could also contribute to the observed differences in prevalence of certain dimensions of burnout syndrome [38].

Several studies have identified an association between alcohol use and burnout syndrome [29,39]. In a national cohort of medical students in the USA, high levels of burnout syndrome domains emotional exhaustion and depersonalization were strongly associated with alcohol abuse/dependence [39]. Similarly, greater alcohol consumption was associated with higher emotional exhaustion and depersonalization among the first-year family medicine residents in the USA [40]. At the University of Cyprus, medical students who consumed alcohol exhibited a higher level of cynicism and lower level of efficacy compared to those who did not consume alcohol, whereby among those who did consume alcohol increasing consumption was significantly associated with lower efficacy [41]. Among 547 medical students in Kazakhstan, 127 (23.2%) reported alcohol consumption, whereby a significant association was found between alcohol consumption and all dimensions of burnout: for high exhaustion and for high disengagement according to the Oldenburg Burnout Inventory for college students, and for personal burnout, studies-related burnout, colleague-related burnout, and teacher-related burnout according to the Copenhagen Burnout Inventory-Students survey [42]. In the present study, high frequency of alcohol use was the main factor that was independently associated with high levels of all three dimensions of burnout syndrome in medical students (every day consumption, P < 0.05; with a significant upward trend – P < 0.05). Similar to this study, a study on burnout syndrome involving 290 medical students from all six years of study in Serbia reported a statistically significant positive correlation between Emotional Exhaustion and alcohol use, as well as between Cynicism and alcohol use [43]. In contrast, research conducted among British medical students found that higher levels of alcohol binge drinking were significantly associated with higher scores of personal accomplishment [29]. But, this link must be interpreted with a lot of caution because even though some studies pointed to the association between alcohol use and burnout syndrome in medical students, there remains the above stated inconsistency in their results and the fact that some studies did not inquire about the use of alcohol due to alcohol consumption being against law and religion [44,45]. Moreover, variations in how the consumption of different forms of alcoholic beverages was assessed across studies may contribute to the differences between reported findings. In addition, given that the mentioned research employed cross-sectional design, it still remains to be determined whether the association between alcohol consumption and burnout syndrome is unidirectional. Namely, some studies suggest that certain individuals use alcohol as a way of coping with stress [46,47]. On the other hand, research from the UK found that young drinkers have a “hedonic” approach to excessive alcohol consumption, implying that medical students may engage in drinking for pleasure rather than to cope with stress and burnout [48]. While numerous studies have reported frequent alcohol use among medical students [41,49,50], this finding is not universal [42]. According to the National Health Research Study conducted in Serbia in 2013, approximately 1.3% of individuals aged 15–34 reported daily alcohol consumption in the previous 12 months [51]. In Serbia, drinking alcohol is considered socially acceptable (as part of traditions and customs), which results in a high prevalence of alcohol use, particularly among young people. A recent systematic review showed that alcohol and illicit substance use were strongly related with burnout syndrome in medical students [44], but emphasized that temporal associations could not be confirmed, due to the risk of reverse causality. These temporal associations can be verified by conducting epidemiological studies with a longitudinal design, to clarify whether alcohol consumption directly leads to burnout syndrome in medical students or whether high levels of burnout lead to resorting to alcohol use.

In this study, sedative use was significantly associated with high level of emotional exhaustion and cynicism among medical students. According to French authors, medical students exhibit a high prevalence of substance use, though the underlying motives remain unclear – whether such behavior is due to attempts at self-medication (e.g., in order to cope with stress) or due to motives such as pleasure or looking for something new [52]. A study in India, which surveyed medical students at the beginning and at the end of their first study year, reported significant increases in depression and stress but not in burnout [53]. Some studies have noted that rates of depression are higher among medical students compared to the general population, however they are not more likely to seek treatment – only 22% of students experiencing depression reportedly pursue help, due to concerns about confidentiality, stigma or cost [54–55]. It remains an open question whether the use of drugs (such as antidepressants, anxiolytics, or sedatives) serves as an indicator of the presence of these basic pathologies, which may in turn make the occurrence of symptoms of burnout more likely. It is unclear whether the use of sedatives directly causes more burnout in students, or whether those students already experiencing high levels of stress resort to recreational drugs for comfort [56,57]. Data on the use of psychoactive substances were provided by a total of 8 (1.1%) respondents – 5 students with burnout (4.3% of those with burnout) and 3 students without burnout (0.5% of those without burnout). This did not allow specification or breakdown by type of the substance used, leading to the loss of interpretability of these results. Interpreting the relationship between risk factors such as sedative use and alcohol use and burnout syndrome in medical students requires careful consideration of bidirectionality, since burnout can also trigger sedative and alcohol use. Current studies, like most research on burnout syndrome and substance and alcohol use, employed cross-sectional design and cannot confirm causality among the examined variables [44]. Longitudinal studies are needed to establish a clear understanding of causal relationship and clarify the direction of influence between burnout and alcohol and substance use. Given that education on drug abuse, alcohol use and illicit substances represents a core component of the medical schools’ curriculums [58–60], this association should be explored further in future longitudinal studies.

According to the findings of this study, high level of emotional exhaustion was significantly increased with the clinical training level of studies in medical students. Even though these results align with many prior studies showing that medical students have relatively low burnout rates when enrolling the medical school but experience a significant increase in emotional exhaustion upon exposure to clinical courses [61–63], some authors have reported different findings [38,64]. Contrary to this research, another study on burnout syndrome among medical students of the first and fifth year of studies in Serbia observed statistically significant differences in the values of all three burnout subscales in those students, with MBI EE being higher among first-year students [21]. A study conducted in Brazil recorded higher scores for emotional exhaustion, depersonalization and personal accomplishment among medical students in final stage of their studies [65]. The transition from preclinical to clinical training can be associated with factors such as emotional load due to moving from classroom to clinic, and educational pressure anxiety which is related to performing clinical skills, fear of unintentional harm to patients, wrong treatment, concerns of risk of infections from patients and loss of control over personal time, particularly sleeping hours. Research showed that rates of depression, anxiety and psychological distress are higher in the higher years of studies compared to the general population [66]. In a study conducted in Trinidad and Tobago, medical students had the highest levels of burnout and depression in the final year of study [67].

In this study, a positive personal medical history (presence of any chronic disease) was frequently reported among medical students who had high risk of academic inefficacy. However, only a few studies have examined the relationship between mental and physical health of medical students and burnout [26]. At an academic health center in the USA, both ever diagnosed physical and mental conditions were more associated with higher burnout among medical students, faculty and staff [68]. Regarding the personal accomplishment subscale, significant differences were observed only in relation to psychological health among fourth year medical students at the University of South Africa [69]. A cross-sectional study conducted across 22 medical schools in Brazil found significant associations between personal accomplishment and psychological quality of life and reduced personal distress [65]. The burnout–disease relationship can be regarded as part of a process in which academic demands compromise students’ mental and physical resources, leading to consequences such as academic inefficacy [58].

This study demonstrated significant correlations between medical student characteristics and dimensions of burnout, with the most prominent determinant being high frequency of alcohol use (every day consumption, P < 0.05; with a significant upward trend – P < 0.05). The findings of this study have important implications for student support services, suggesting counsellors should provide useful educational and psychological advice to all medical students, particularly students who are at high risk for burnout. The considerable variability in reported frequency of burnout among medical students can be attributed to the use of different questionnaires for burnout assessment, use of different criteria for identifying the syndrome (in addition to the three-dimensional – the use of one- and/or two-dimensional criteria) by researchers, and the application of non-validated instruments instead of validated ones [11]. For research in the student population, additional longitudinal studies that should use defined criteria and standardized instruments, adhering to a rigorous scientific approach, are necessary.

During the period of online learning necessitated by the COVID-19 pandemic, the prevalence of burnout syndrome in medical students ranged from 16.7 to 59.9% [70]. Available evidence indicates that medical students encountered numerous challenges during distance learning [71–73]. Circumstances that had an impact on the psychological well-being of medical students included transition to distance learning, reduced contact with peers, impaired transfer of knowledge, particularly for clinical courses, and often additional responsibilities such as volunteer work with patients with COVID-19 [71–76]. Throughout medical school, students generally exhibited a trend of consistently high scores on the personal accomplishment and emotional exhaustion subscales, along with an increasing trend on the depersonalization subscale [70,74–77]. In addition to burnout syndrome, medical students during the COVID-19 pandemic reported elevated levels of stress, anxiety, depression, sleep disturbances, lack of social support, suicidal ideation, and lack of physical activity, all of which have been related to burnout [75–77]. To effectively address and prevent these problems in the context of potential future health crises, medical schools should take proactive measures to support students, which include counseling students by promoting mental well-being and healthy lifestyle, planning interventions to prevent burnout syndrome – such as coping strategies and self-motivation, and integrating them into the curriculum. Key findings indicate the need for the development of health-related guidelines, which should include monitoring quality of life of medical students, particularly during the clinical courses, and actively promoting a healthy lifestyle which involves regular exercise, adequate sleep, stress management skills, and a positive learning environment [72,76,78,79].

### Strengths and limitations of the study

Despite the high response rate and the use of a validated questionnaire, several limitations may have affected the findings of this study. Notably, while the MBI was applied according to the traditional use of cut-off points, it is important to acknowledge that the most recent MBI manuals recommend avoiding cut-off points when interpreting MBI results and treating burnout as a continuous variable/phenomenon, and this change in approach is a potential source of limitation in the current study [35–37]. First, due to the well-known shortcomings of cross-sectional studies, the causality cannot be attributed to the associations between burnout dimensions and medical students’ characteristics observed in this study. Second, this study was conducted at a single medical faculty study, which may limit the generalizability of findings. Additionally, the question of sample size can always be raised. Furthermore, data on academic performance and other student characteristics are based on self-reporting by medical students, without any insight or verification in their relevant documentation. A significant limitation of this study is its reliance on data collected in 2014, which may reduce the applicability of some of the original findings of the study for understanding the contemporary situation. Finally, the possibility of bias (informational and/or response) cannot be excluded. In addition, it cannot be disregarded that the burnout dimensions are related to some other characteristics (such as socio-economic status, etc.) that were not examined. Therefore, the findings should be confirmed in future research or using longitudinal studies.

## Conclusions

This study highlights the relationship between the dimensions of burnout syndrome (i.e., high level of emotional exhaustion, cynicism, and academic inefficacy) and certain characteristics of medical students. The identification of modifiable factors that are associated with burnout in Serbian medical students underscores the need for targeted support that would provide medical students the necessary help in order to prevent burnout syndrome. Since this study was conducted at a single Faculty of Medicine in Serbia, the findings cannot be properly generalized, indicating the need for further research involving a national sample of medical students.
